# 147. Clinical impact of the BioFire® FilmArray® Pneumonia (PN) Panel in the Veteran Affairs healthcare system using a difference-in-difference (DiD) design

**DOI:** 10.1093/ofid/ofaf695.049

**Published:** 2026-01-11

**Authors:** Nicholas Britt, Karim Khader, Tao He, Andrea M Prinzi, Benjamin Hommel, Tristan T Timbrook, Thomas Lodise

**Affiliations:** University of Kansas School of Pharmacy, Kansas City, Kansas; University of Utah, Salt Lake City, Utah; Veterans Affairs Salt Lake City Health Care System, Salt Lake City, Utah; bioMerieux, Salt Lake City, Utah; bioMerieux, Salt Lake City, Utah; Barnes-Jewish Hospital, Saint Louis, Missouri; Albany College of Pharmacy and Health Sciences, Stratton, VA, United States, Stratton, VA

## Abstract

**Background:**

The BioFire® FilmArray® PN Panel (BioFire) improves time to pathogen detection, but real-world evidence of clinical impact is limited. This study evaluated the clinical outcomes associated with BioFire implementation in ICU patients within the US Veterans Affairs (VA) system using a quasi-experimental design and difference-in-differences(DiD) analysis.

Comparison of Baseline Characteristics Between BioFire® and Control Groups

**Figure:**
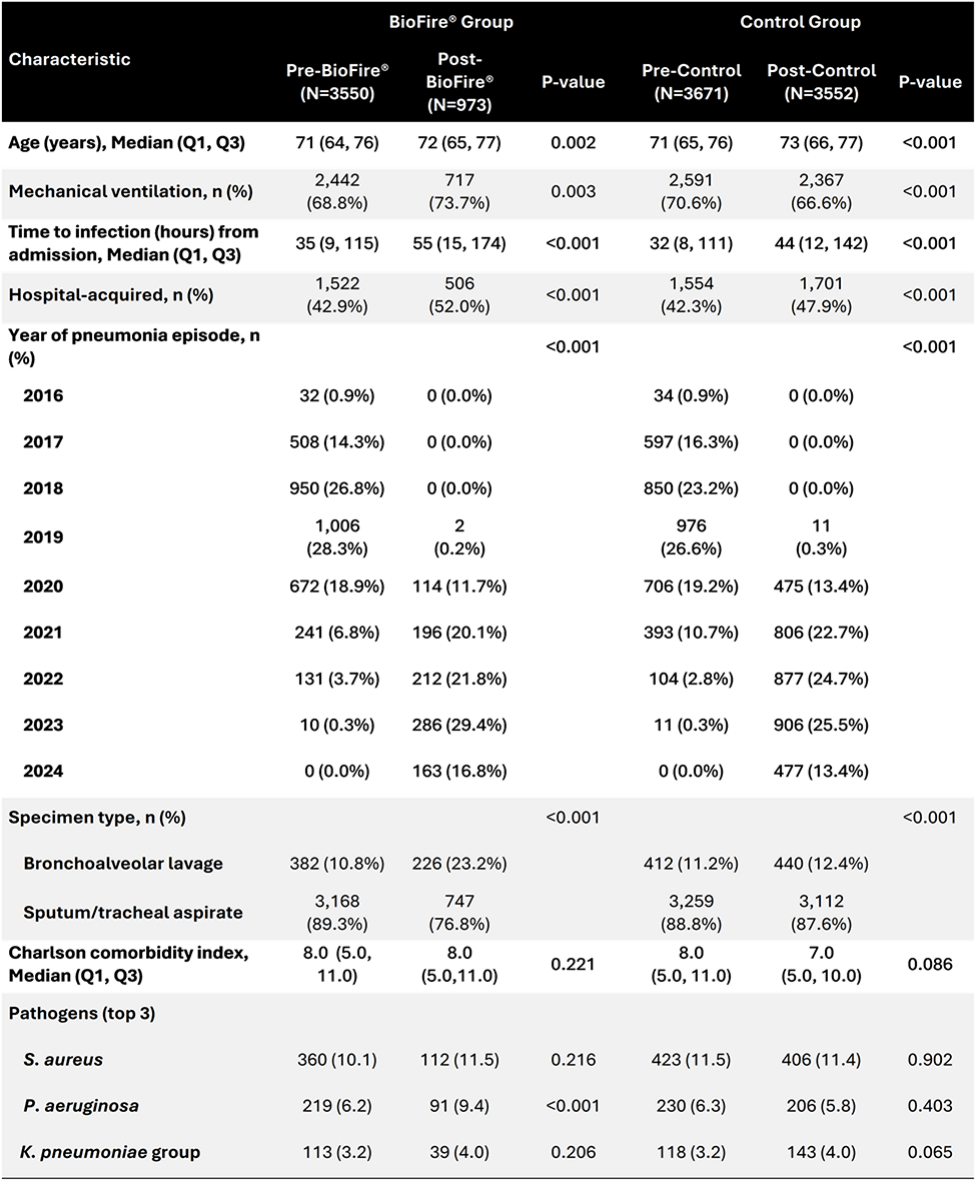

Unadjusted Differences in Outcomes between the BioFire and Control groups in the Pre-Post BioFire Implementation Periods Overall and Among Patients with a Positive Result for a BioFire PN Panel Bacterial Pathogen

**Figure:**
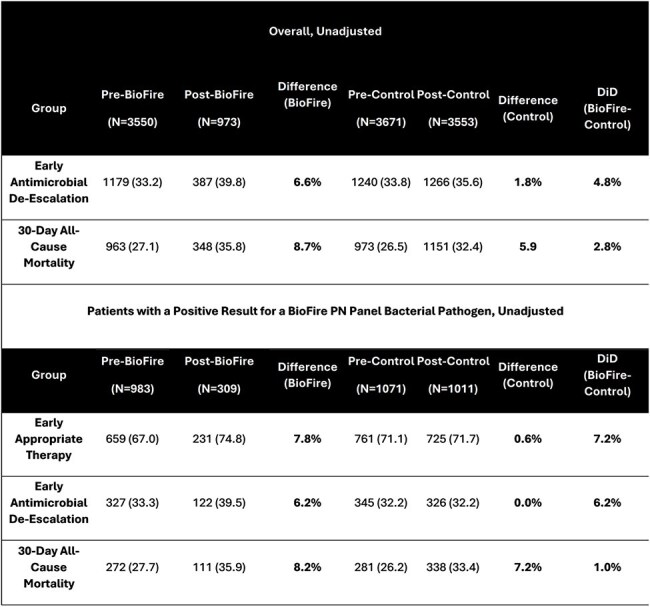

**Methods:**

A retrospective, multicenter, quasi-experimental study was conducted in the VA system (2016–2024). Hospitals implementing the BioFire were matched 1:1 to non-BioFire (Control) hospitals by time, complexity, and geographic region. Patients were first stratified by group (BioFire vs. Control) and implementation timeframe (Pre vs. Post) and were included if they had an ICU admission, radiographic evidence of PN, respiratory culture performed (and BioFire test in Post-BioFire group), survival ≥24 hours after index PN culture, and receipt of ≥1 antibiotic within 2 days of index PN culture. Outcomes included early antibiotic de-escalation (reduction in spectrum score < 24 h of index culture), early appropriate therapy (receipt of appropriate therapy < 24 h of index culture/Biofire test), and 30-day all-cause mortality. The effect of BioFire implementation was estimated using a DiD Poisson regression model, with and without inverse probability of treatment weighting (IPTW) adjustment overall and among patients with a positive result for a BioFire PN Panel bacterial pathogen on a BioFire PN Panel test or respiratory culture.

**Figure:**
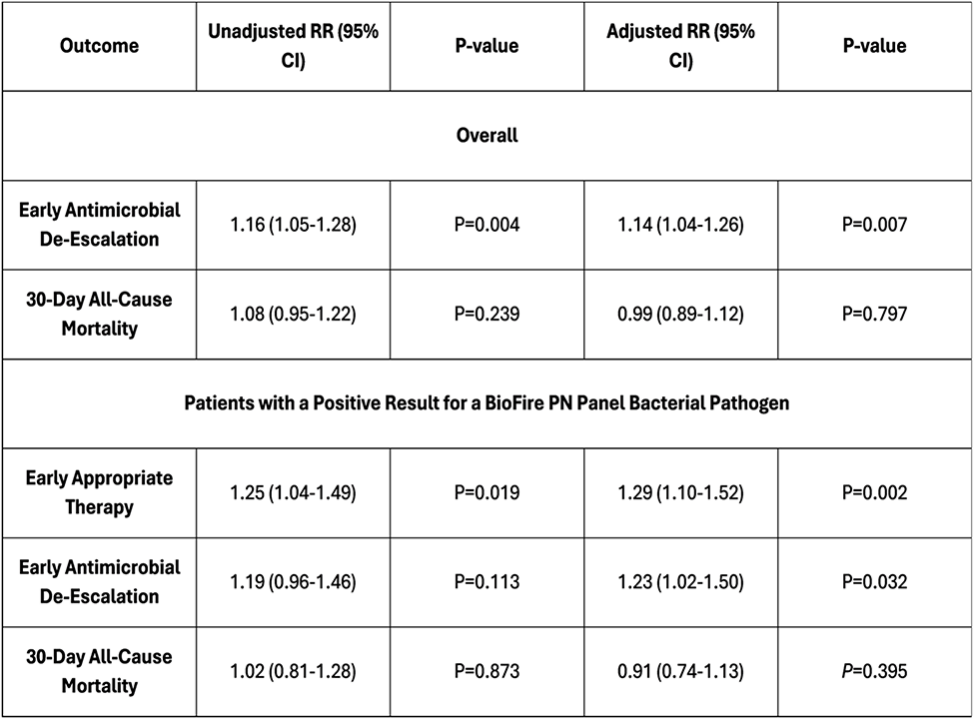
Clinical Outcomes Associated with BioFire® Implementation in Difference-in-Difference Poisson Regression Analyses

**Results:**

A total of 11,746 patients across 36 facilities met study criteria. Many significant baseline differences were noted pre- vs. post groups, while Pre-BioFire-Pre-Control and Post-BioFire-Post-Control were similar (Table 1). Unadjusted outcome differences in outcomes between the BioFire and Control groups are shown in Table 2. In the DiD regression analyses (parallel trends assumption was met), BioFire implementation was associated with improvements in receipt of early appropriate therapy and de-escalation, but not 30-day mortality in overall analyses. Similar results were observed in patients with a positive BioFire/culture result (Table 3).

**Conclusion:**

BioFire PN Panel implementation was associated with improved antimicrobial optimization in ICU patients with PN in the VA system.

**Disclosures:**

Thomas Lodise, Jr., PharmD, PhD, GSK: Advisor/Consultant

